# Women’s responses to changes in U.S. preventive task force’s mammography screening guidelines: results of focus groups with ethnically diverse women

**DOI:** 10.1186/1471-2458-13-1169

**Published:** 2013-12-12

**Authors:** Jennifer D Allen, Shirley Morrison Bluethmann, Margaret Sheets, Kelly Morrison Opdyke, Kathryn Gates-Ferris, Marc Hurlbert, Elizabeth Harden

**Affiliations:** 1 Dana-Farber Cancer Institute, 450 Brookline Ave, Boston, MA 02215, USA; 2 Harvard Medical School, Boston, USA; 3 University of Texas School of Public Health, Houston, USA; 4 Cicatelli Associates Inc. (CAI), New York, USA; 5 Avon Foundation for Women, New York, USA

**Keywords:** Mammography, Screening guidelines, Health communication

## Abstract

**Background:**

The 2009 U.S. Preventive Services Task Force (USPSTF) changed mammography guidelines to recommend routine biennial screening starting at age 50. This study describes women’s awareness of, attitudes toward, and intention to comply with these new guidelines.

**Methods:**

Women ages 40–50 years old were recruited from the Boston area to participate in focus groups (k = 8; n = 77). Groups were segmented by race/ethnicity (Caucasian = 39%; African American = 35%; Latina = 26%), audio-taped, and transcribed. Thematic content analysis was used.

**Results:**

Participants were largely unaware of the revised guidelines and suspicious that it was a cost-savings measure by insurers and/or providers. Most did not intend to comply with the change, viewing screening as obligatory. Few felt prepared to participate in shared decision-making or advocate for their preferences with respect to screening.

**Conclusions:**

Communication about the rationale for mammography guideline changes has left many women unconvinced about potential disadvantages or limitations of screening. Since further guideline changes are likely to occur with advances in technology and science, it is important to help women become informed consumers of health information and active participants in shared decision-making with providers. Additional research is needed to determine the impact of the USPSTF change on women’s screening behaviors and on breast cancer outcomes.

## Background

Research has shown that having a mammogram can play a critical role in early detection and treatment of breast cancer [1], the most common cancer diagnosis among women worldwide [2,3]. Routine screening with mammography has been a focus of cancer control efforts for the past quarter century. Recommendations to begin routine screening for women at age 40 have been in place in the US for two decades [4]. However, the appropriate age to initiate and intervals to continue routine mammography screening have generated controversy across the globe.

Internationally, debate has surged around benefits of mammograms against potential harms, most notably related to “over-diagnosis”. A recent meta-analysis conducted by an independent panel of international scientists estimated a 20% reduction in breast cancer mortality in women that participated in screening over a 20-year period based on data available from 11 randomized controlled trials as well as observational studies [5]. However, based on this data, they also estimated that 19% of the cancers identified in these patients may have constituted over-diagnosis of cancers that would not have come to clinical attention in those women’s lifetimes [5]. At present, the UK National Health Service Breast Screening Programme invites women ages 50–70 years for routine triennial screening, but women within three years of this age range (47–49 and 71–73) are also eligible to participate in screening through self-referral [6].

In 2009, the US Preventive Services Task Force (USPSTF) reviewed available evidence and recommended that healthy women begin routine biennial mammograms at age 50, a change from the previous recommendation to begin routine annual mammograms at age 40 [7]. The most compelling evidence in this decision came from comprehensive review of epidemiological studies which showed limited long-term reduction of late-stage cancers in countries with similar screening practices; a tendency to “over-diagnose” by 20-40% changes in breast tissue of younger women being screened; and unnecessary radiation exposure for women younger than 50 years [8-12]. Under the new recommendations, US women younger than age 50 are advised to make individualized screening decisions in consultation with their healthcare providers, after considering the potential benefits and harms of screening (‘informed decision-making’) [7]. While these guidelines are supported by many major US medical organizations [13], debate persists in the scientific community, especially as other major cancer authorities, such as the American Cancer Society, continue to recommend routine annual mammograms beginning at age 40 [14]. These conflicting recommendations have contributed to a climate of confusion for both providers and patients [15-17].

Researchers have begun to assess the effects of this debate on public perceptions about mammography [18]. Early reports have suggested that the new guidelines have had little impact on mammography rates among women ages 40–50 [19]. In a randomized controlled trial in which 249 women 39–49 years were exposed to two types of media reports around the 2009 guideline changes, 84% said they would *not delay* routine screening exams to age 50, even if their doctor made that recommendation [20]. Women who had experienced a prior false-positive mammogram were found to be less likely to adhere to the new guidelines and to over-estimate their lifetime risk of breast cancer [20]. Some researchers contend that women have been more receptive to past recommendations because they focused on earlier and more frequent screening [21] – a by-product of what some have called an “enthusiasm for cancer screening” in the US [22]. But, generally, women and physicians report low screening knowledge and low engagement in shared decision-making about breast cancer screening [23-25].

Recent studies suggest a need for greater exploration of the impact of changes in screening recommendations, and on how decisions related to mammograms are made [26,27]. This may be especially important for minority and immigrant women, for whom cultural factors may shape attitudes and beliefs about the importance and harms of breast cancer screening [28,29]. Despite recent studies that affirm persistent disparities in screening access and treatment for breast cancer, [30-33] the USPSTF guidelines do not address racial/ethnic differences in breast cancer incidence or mortality, leaving room for debate on their applicability to specific populations [7,34]. There is also a lack of empirical data to assess the impact of recent policy changes on mammography uptake [35,36].

Understanding the extent to which women are aware of guideline changes, the controversy that has erupted, and how they reconcile inconsistent messages from a variety of information sources is critical to developing interventions that can improve decision-making processes related to breast cancer screening. A few recent studies have begun to assess attitudes, knowledge and potential interventions for appropriate use of mammograms [37,38]. Yet more specific exploration of communication needs, especially for diverse women, is needed. This qualitative study explored knowledge, awareness and attitudes about breast cancer screening and the USPSTF guideline changes among Latina, African American and Caucasian women. Discussions also assessed women’s information needs, decision-making processes, and preferred channels and sources of information delivery.

## Methods

We selected focus group methodology for our qualitative data collection, as it offers several advantages over other methods. With this method, participants share a range of perspectives, allowing each to consider the extent to which her experience is similar to or different from those expressed by the others. This process can facilitate a more thorough exploration of an individual’s own experience. Also, focus group methodology makes the researcher a less commanding or controlling presence, thereby allowing participants to more easily express their opinions and perceptions. As a result, the discussion may uncover themes that could shed new light on key aspects of the phenomena of interest. Finally, the collective context of focus groups enables pertinent themes to arise more quickly than they might during individual interviews [39,40].

Our focus group discussion guide consisted of a list of open-ended questions (Table 1) based on our previous work [41-43] and the few existing studies of women’s reactions to changes in cancer screening guidelines [18,20]. Data were collected between February and April 2011. Eligible women were between the ages of 40–50 and were English or Spanish speaking. Women (n = 77) were recruited through word-of-mouth, fliers, and by personal contact with research assistants in community and social service agencies, housing developments, and churches in the Greater Boston metropolitan area. Written informed consent was obtained from the participants prior to participation. Focus groups took place at YWCAs in three communities and at a community health center. Data collection ceased at the point of saturation, that is, when no new themes emerged.

**Table 1:** Focus group questions

**Construct**	**Sample question**
General health	What health issues are you most concerned about, for yourself?
	What do we know about the causes of breast cancer? What have you heard?
	What do women do to prevent breast cancer?
	How do you find out if you have breast cancer?
	What do you think is the most important thing women can do to detect breast cancer early?
	*If no one mentions mammograms, ask*, “Have you heard about mammograms? What have you heard?”
Mammograms	When should you get your first mammogram? At what age?
	How often should you have a mammogram after that?
	Who makes these recommendations?
	Have you seen or heard anything recently about changes in these recommendations?
	How did hearing or seeing this information make you feel?
Screening guidelines and changes in screening guidelines	What are your thoughts and concerns about these changes?
	Why do you think the guidelines changed?
	What makes you think the guidelines changed because of < *insert participants’ answer* > ? (*probe*)
	How does the change make you feel?
	Do you think the guidelines will change again? Why or why not?
	How would you feel if the guidelines changed again?
	How do you think the change in the mammography screening guidelines will affect you?
Decision making	Think about a time when you had to make a health decision for yourself or some one in your family. Did you want to talk about it with your health care provider?
	What made you want to discuss it with your provider?
	What did you want to know?
	How did your provider respond?
	How did you feel about that response?
	What would have made that experience more successful for you?
Education	What do you think is the best way for women to learn about this issue, making decisions about mammograms?
	*Probes:*
	From their providers
	Health education materials (from where? Insurer?)
	Provider? News outlets? Online?
	Peer health leaders or navigators
	What do you think are the 3 most important things women in their 40s need to know?

Groups were segmented on race/ethnicity because prior studies have shown that these groups may have distinct beliefs and attitudes about screening [44,45]. There were 8 groups with 6–10 women in each. Trained moderators were matched according to the race/ethnicity and language of each group and conducted the groups according to a standardized protocol. Each group discussion was audiotaped, professionally transcribed and where necessary, translated. A note-taker was present to document non-verbal communication and observe participants’ levels of participation. Each session lasted between 60–90 minutes and participants each received $50. All study protocols and procedures were approved by the Institutional Review Board at the Harvard School of Public Health.

### Analysis

Constant comparative analysis of transcripts was conducted, based on procedures outlined by Strauss and Corbin [46]. Three of the authors (JDA, SMB, MS) independently reviewed each transcript using open coding to create categories. Subsequently, the investigator team reviewed the categories and supporting quotes to develop an initial coding scheme. Through this process, open codes were collapsed into higher order categories that reflected emergent themes. When theme interpretations differed, the transcripts were reviewed further and discussed until consensus was achieved [47].

## Results

### Characteristics of participants

We conducted eight focus groups with a total of 77 participants. As shown in Table 2, 35% were African American, 26% Latina and 39% were Caucasian. Two of the focus groups were conducted in Spanish. The mean age of participants was 44.6 years.

**Table 2:** Socio-demographic characteristics of focus group participants

**Characteristic**	**N (77)**	**(%)**
Age (mean, sd)	77	44.6 (4.3)
Educational Level		
< HS	5	6.5
HS or GED	16	20.8
Some College or technical school	32	41.6
College graduate or higher	24	31.2
Annual Household Income		
< Less than $10,000	12	15.6
$10,000 $29,999	16	20.8
$30,000 - $49,999	24	31.2
More than $50,000	20	26.0
Don’t know/not sure	4	5.2
Missing	1	1.3
Insurance status		
Insured (private)	38	49.4
Insured (public)	23	29.9
Insured (type missing)	7	10.4
Not insured	8	9.1
Missing	1	1.3
Race/Ethnicity		
White, non-Hispanic	30	39
Black, non-Hispanic	27	35
Hispanic	20	26

### Themes

Following the multi-stage coding process, we grouped predominant themes into three categories: (1) knowledge, awareness and attitudes toward mammograms and the USPSTF guideline change; (2) decision-making with respect to breast cancer screening; and (3) preferences regarding channels and sources for information. See Table 3 for predominant themes and supporting quotes.

**Table 3:** Themes and sample quotes

**Theme**	**Sample quotes**
**Knowledge, awareness & attitudes toward mammography screening guideline changes**	
Low levels of knowledge & awareness; confusion	o “I think that we, as Hispanic women, sometimes have little knowledge. That’s why we are the most exposed to cancer, to die from it. So many people lack the right information.” (Latina)
	o “The guidelines have changed without us even knowin’, evidently. Because we don’t know about it.” (African American)
	o “I’m very confused by that because of, you know, I think it was about a year ago …they kind of changed what they were saying…” (Caucasian)
	o “If my doctors tell me that I still need it every year, is my insurance then telling me I’m not?… it just added confusion, that STILL isn’t resolved.” (Caucasian)
Belief that screening more often & at younger ages is best	o “I don’t understand why do we get our first mammogram after 40, if nowadays there is so much cancer among younger people.” (Latina)
	o “There are so many cases of women (with cancer). I don’t even know why we are discussing (raising age of first mammogram). It is not logical to me.” (Latina)
	o “Since there is cancer in people who are so much younger, starting at 50 or asking the doctor at 40 doesn’t make sense.” (Latina)
	o “In our ethnic group, we already have women diagnosed with breast cancer in their 20s. Why not start early.” (Latina)
	o “[Yearly mammogram have] been drilled in our head for years and years and years and then, all of a sudden you’re just changing it, just like that.” (Caucasian)
Mistrust regarding rationale for change	o “I really think that that comes from the insurance companies… (who will not) want to pay for an exam every year.” (Latina)
	o “Insurance companies tells doctors what to do.” (African American)
	o “This is something someone told me long ago; the notion that (current screening guidelines are) not cost-effective…I am sorry. Life is more important than ‘cost-effective.’” (Latina)
	o “… the sense I got was, ‘Okay, they’re belittling us, that we can’t handle it… you know, the false/positives.’ Then my interpretation was, ‘Well, it’s cost-cutting’ … what I was hearing… didn’t [make] sense.” (Caucasian)
	o “They’re trying to save a dollar.” (Caucasian)
Excluded from decision-making & policy process	o “Who asked me?…Did they ask women how they would feel if it was changed to 50? Or did they change it first and now they are asking me after the fact?” (Latina)
	o “Is… the government trying to dictate when a woman needs a screening test?” (Latina)
	o “I don’t accept [the change] it because I feel that it should have been…told to everybody …there’s a lot of people in here and only one person heard about it, the word has to get out… women need to know what’s goin’ on!” (African American)
	o “It was just, ‘Here’s the change, now do it this way.’ Without any explanation.” (Caucasian)
	o “It was like, ‘Well, they’re not gonna know better,’ type of thing. They’re just women. It was just men, deciding FOR us. I felt very diminished, the way they were talking about me [about] women in general.” (Caucasian)
**Factors that influence decisions to undergo breast cancer screening**	
Not a decision	o “I would still want my mammogram done, no matter what. I’m forty-seven and I’ve had problems in the past…I would still like to be on the safe side.” (African American)
	o “(If my doctor told me not to have a mammogram) I would think somethin’ is wrong with her. Why is she tellin’ me NOT to have it done? Shouldn’t it be my choice?” (African American)
	o “(If the doctor told you not to have a mammogram), I would just say, ‘I don’t feel comfortable, and I just feel like I need to have one.’” (African American)
	o “I would tell the doctor that if he is not willing to refer me to do the (mammogram) that I’ll go somewhere else. Because I’m worried…” (Latina)
“Following doctor’s orders”	o “The doctor decides whether or not you get a mammogram.” (Latina)
	o “In my case, I didn’t have to (schedule my mammogram) myself, because they, my doctors, take care of it and refer me to the appointment every year…But if they didn’t do it, I would.” (Latina)
	o “I got my mammogram three times and the last two times, they told me I had a cyst on one of my breasts. So, I asked them, ‘what are you going to do to remove it? Am I going to get surgery?’…(The doctor) told me…only if I wanted to. And I think that’s wrong. They have to do what needs to be done…Not leave it up to me.” (Latina)
	o “Well, you know what I noticed? A lot of women in our community, right here, are not educated enough to know that they can (decide to have a mammogram on their own)…they just go by what their doctor says.” (African American)
Need to advocate for oneself	o “A lot of women in our community, right here, are not educated enough to know that they can (decide to have a mammogram on their own)…they just go by what their doctor says.” (African American)
	o “I wouldn’t follow the guidelines. (I’d still get a mammogram) once a year.” (African American)
	o “Do your research, ask your questions, it’s you and you only have one body, one life.” (African American)
	o “I think women stood up and said, ‘No, you’re not gonna change. This is our health.’” (Caucasian)
**Preferred sources & channels for information delivery**	
Doctors are preferred information source but many have poor patient/provider communication	o “I told (the doctor) I was still concerned about a spot (on my breast). But the doctor made a dismissing (sic) gesture and said, ‘You don’t need an ultrasound. You just need a mammogram. You are fine. I don’t see any spot,’ as if it were unimportant.” (Latina)
	o "Don“t sit back and think that the doctor’s gonna go through all this and that because, you know, the doctor’s mind might be somewhere else…” (African American)
	o “What do I think doctors will do? Some doctors will be COMPLETELY disrespectful of their patient’s thoughts in this situation and say, ‘You don’t know anything. What do YOU know?’” (African American)
	o “I would be interested in potentially making that decision or talking to my doctor.” (Caucasian)
More information & explanation needed; help women make informed decisions & advocate for their preferences	o “If my doctor told me not to have a mammogram, I would think somethin’ is wrong with her. Why is she tellin’ me NOT to have it done? Shouldn’t it be my choice?” (African American)
	o “The thing that bothers me about this, it says, ‘women who are less than 50 have to decide if they want.’ Okay. So, first of all, HOW they decide is really important; who helps them decide -- I’m worried about that.” (African American)
	o “The medical professionals need to be more proactive in helping women understand… especially in the Black community. All of those screenings should be discussed, but they’re not....” (African American)
	o “I’m thinking, ‘Gee, what ARE the cons?’ … But, that discussion didn’t happen. They always ask, ‘Do you have questions?’ But I didn’t get the idea that it was up for discussion. It was kind of like, ‘I’m gonna write you a referral for your mammogram.’” (Caucasian)
	o “You NEED that information from people that you trust so that you can make a decision.” (Caucasian)

#### ***Knowledge, awareness and attitudes regarding USPSTF guideline changes***

The majority of participants were unaware of any change in mammography screening guidelines; only two or three women in each focus group reported knowing of the change. The others were unaware of the recent debate within the medical community about the appropriate age and interval for mammography screening, although some noted that they had heard about this in the past (referring to earlier guideline changes). When informed about the recent change, many expressed disbelief and confusion. One participant said, *“I don’t understand why we (shouldn’t) get our first mammogram til after 40, if nowadays there is so much cancer among younger people” (Latina).*

A suspicion held by many women was that the guideline changes were motivated by cost-cutting measures. For example, *“I really think that [the change] comes from the insurance companies. They do not want to pay for an exam every year”* (Latina). Others noted, *“Insurance companies tell doctors what to do”* (African American) and “Yes. That’s what I think. I just think it’s the insurance companies. Sorry” (Caucasian). Discussions in each of the groups involved the notion that insurance companies were not acting in women’s best interests. Many expressed fears that under the new USPSTF guideline, insurance companies would no longer cover mammograms for women under 50.

Other participants expressed feeling betrayed by the process for revising the mammography recommendations, since they believed that women should have been consulted before the new guidelines were established. For example, one participant asked: *“Who asked me? Did they ask women how they would feel if it was changed to 50? Or did they change it first and now they are asking me after the fact?”* (Latina). Others expressed the opinion that it was paternalistic for presumed “experts” to decide which would result in greater harm, over-detection and diagnosis or heightened anxiety and fear. One woman commented, “*I was…almost MAD at the news…they’re belittling us, that we can’t handle it…you know, the false positives,”* (Caucasian). Multiple women in the Caucasian groups said they strongly believed in the importance of mammograms for early detection and “protection” against breast cancer. A theme that was particularly apparent among the focus groups with African American women was that the guideline change signified withdrawal of access to an important, potentially “life-saving” procedure.

Although there was consensus for at least an annual mammogram, there were frequent comments regarding the fear, discomfort and anxiety that accompanied breast cancer screening. Women shared stories about missed diagnoses, false positive findings, and painful diagnostic procedures. Still, most said that they would continue with routine screening. Indeed, many said that they wanted to be screened more than once a year and to start at a younger age (<40).

#### ***Decision-making with respect to breast cancer screening***

Given the low levels of knowledge about the guideline change, many women acknowledged that they simply “followed doctor’s orders,” rather than taking part in decision making. One participant stated*, “A lot of women in our community. . .are not educated. . .they just go by what their doctor says”* (African American). Others commented that they would defer to the doctor’s recommendation, even if it conflicted with their own preference. “*I assumed (getting a mammogram) was best for me, and that’s what I did. I didn’t question it. So if that means I’m gonna have to pay for it, I will take care of myself and I will have it done. I thought that was the best thing to do,*” (Caucasian). A smaller number of women expressed support for shared decision making *“The decision to have a mammogram is between you and your doctor. . ”.* (African American).

Mistrust about the revised screening guidelines was often accompanied by discussion of how women often leave decisions to their providers. The juxtaposition of these two themes led to lengthy discussions about the need for women to become better advocates for themselves. Although few women reported that they felt confident taking an active role in medical decisions *themselves,* many said that they would strongly urge their peers to be more active in the decision-making process. One respondent advised, *“Do your research, ask your questions; it’s you, and you only have one body, one life”* (African American). Some women also felt that women younger than 50 should be educated about their “right to be screened”. This was evident in the quote from one woman who stated, “*We want to make sure that if you’re younger than 40 AND you feel like that’s what you need [a mammogram] . . . there needs to be a way . . . to get [one]. It’s not just a ‘NO, you can’t get it”* (African American). The notion that one needed to be prepared for a discussion of mammography with one’s provider was particularly prevalent among African American women; Latinas were less likely to express a need to prepare for the discussion and more likely to report that they would defer to the providers’ recommendation.

Learning about the revised guidelines seemed to strengthen rather than diminish women’s commitment to mammography screening. Quotes such as*, “I won’t follow the guidelines. I’d still get a mammogram once a year,”* (African American) were common. Others suggested the doctor would be negligent if he or she did not recommend screening, for example, *“If my doctor told me not to have a mammogram, I would think something is wrong with her,”* (African American). Another said that she would not trust a doctor who told her not to be screened, saying: *“I would tell the doctor that if he is not willing to refer me to do the (mammogram) that I’ll go somewhere else”* (Latina).

#### ***Preferences regarding channels and sources for information***

Although most participants cited their medical providers as their preferred source of health information, most expressed dissatisfaction with the frequency and quality of conversations with them. Many also shared experiences of having doctors minimize their concerns: *“I told (the doctor) I was still concerned about a spot (on my breast). But the doctor made a dismissing gesture and said, ‘You don’t need an ultrasound. You just need a mammogram. You are fine. I don’t see any spot,’ as if it were unimportant”* (Latina). Regarding mammography, one woman expressed concern that many women aren’t able to stand up for themselves and express their preferences to their providers. She stated, *“I’m gonna keep standing for women who don’t have that opportunity [to express their preferences], cause I have BEEN in that place! What do I think doctors will do [if women want a role in decision-making]? Some doctors will be COMPLETELY disrespectful of their patients’ thoughts in this situation and say, ‘You don’t know anything.“ What do YOU know?”* (African American)

Among the Latina focus groups, the influence of family members’ opinions was clearly evident. There were multiple stories about receiving health advice from mothers or grandmothers. One participant stated, *“… Latino women rely a lot on what our grandmothers taught us: ‘In case of any pain, you take a hot tea.’”* Another shared: *“Latino women rely on information/folk cures shared through generations”.* When asked specifically about making a decision about whether or not to have breast cancer screening, one woman stated: *“In a situation like that, what you need the most is the support from your family”.* (Latina).

When presented with the idea of a print brochure to assist with informed decision-making about mammography—including information about both pros and cons of screening-- women were divided. Most expressed that they would have “no use” for a decision aid because they planned to get screened regardless of screening guidelines. In all of the groups, women expressed skepticism about the information regarding disadvantages of mammography. Multiple Caucasian women said that they needed mammograms because they believed “early detection is everything” and mammograms were central to this belief (Caucasian). In other groups, the rejection of the potential disadvantages of mammograms was more blatant. One woman went so far as to say: *“This information is full of [expletive]”* (African American). A few acknowledged that there might be some merit in such an educational brochure, but remained concerned about their ability to sift through inconsistent information about mammography or “go against” what their provider recommended. There was also fear about insurance coverage. One woman put it this way, *“The thing that bothers me about this [brochure], is it says, “Women who are less than 50 have to decide if they want [a mammogram].‘ Okay. So, first of all, HOW do they decide? Who helps them decide? I’m worried about that. Secondly, is it going to be covered if you DO decide [to be screened]?”* (African American).

##### 

**Comparing responses between women of different ethnic groups** In analyzing the data, the authors were careful to ensure that the emerging themes fairly represented women across all three racial/ethnic groups included in the study (Table 3). However, there were some general variations in how these themes related to each racial/ethnic group that may be important to highlight. For example, Caucasian and African American women seemed to be the strongest proponents of retaining the practice of annual mammograms beginning at age 40. Caucasian women, in particular, seemed to place high value on the current culture of breast cancer awareness and perceived role of mammograms in the breast cancer “battle”. African American women were also fervently in support of earlier guideline recommendations partly because they felt familiar. As previously discussed, there was also a resistance to guideline changes among these women because the process by which they were revised did not recognize their part in the decision, a sentiment that was echoed among Latina women. Of all three groups, Latina women seemed least confident about advocating for their own preferences and their ability to participate in shared decision-making.

## Discussion and conclusion

### Discussion

Many women in our sample were not aware of the change in the USPSTF mammography screening guidelines or knowledgeable about the reasons for the policy change. Upon learning of the change, most women expressed strong opposition. There was a deep sense of mistrust regarding the underlying reasons for the guideline change, with many assuming that there was a financial motive among insurance companies, providers, or both to limiting access to screening. Moreover, there was a sense of injustice that the lay public had been excluded from the policy-making process. Many questioned the ability and authority of “experts” to weigh the potential benefits and harms of screening on behalf of individual women.

Many participants perceived that an important service-- one that women cited as essential to breast cancer survival-- had been “taken away” from them. This sentiment was particularly prevalent in discussions among African American women. This finding is consistent with recent literature on medical mistrust that has identified suspicion and lack of support from health care professionals as key factors associated with under-utilization of screening, especially among women of racial/ethnic minority groups [48]. Mistrust of medical providers, researchers and the healthcare system, which is deeply rooted in historical injustices, has been a considerable impediment to receipt of all kinds of health services [49,50].

Given this evidence, it is difficult to predict whether greater involvement with providers would necessarily influence appropriate use of mammograms without significant trust-building efforts.

When asked for their opinions about guidelines, many participants expressed a desire to start screening at age 40 (or before), to decrease the time interval between screenings, in fact, there was some thought that there should be no upper age-limit for screening. Although a key element of the USPSTF recommendation is that women make individualized, informed decisions about mammography in partnership with their health care providers, few felt confident in their ability to do so. A more commonly held view was that a woman should find a place to obtain screening or to seek a different provider if they were advised against screening. Although there was discussion about the need to advocate for oneself, this was not something most women felt comfortable or equipped to do. Nor did they see screening as a decision to be made; the notion of weighing benefits versus harms seemed irrelevant to many women. Participants viewed the potential adverse effects of screening, including over-diagnosis, over-treatment and radiation exposure [51], as minimal and worth the risk if it could save their lives from breast cancer. As one participant stated, ““The benefits of screening WAY outweigh the harms,” (African American).

An unexpected finding was that a substantial number of women in this study felt they were “excluded” from the policy making process. This may have been related to the timing of announcements related to the guideline change, which coincided with news of health care reform, potentially stoking fears that the change was a cost-savings measure [7]. In the future, dissemination of information about guideline changes might utilize community-based participatory strategies, which involve all stakeholders (including women) in the process. Such approaches can foster trust between and be mutually beneficial to policymakers and community members [52]. Even though the review of scientific evidence underlying policy may require a level of scientific expertise that cannot be expected of the lay public, the development of health messages, communication strategies, and consideration of timing of announcements regarding guideline changes could certainly benefit from lay involvement. Such involvement could also go a long way to dispel fears or suspicions [53].

We recognize there are limitations to this exploratory study conducted among a convenience sample. Our goal was not to obtain a representative sample, but rather to hear from women from varied backgrounds and with diverse perspectives [54,55]. Our sample was relatively large for a qualitative study, which gives us confidence that the themes most prevalent in this group could resonate with similar groups of women. We also found that our results, particularly around low awareness and knowledge about the rationale for the USPSTF guideline changes, were consistent with the findings of similar studies [18,39,40], although to our knowledge, this is the first study of its kind to assess awareness across multiple racial/ethnic groups.

We also acknowledge that those who volunteered to participate may have been more interested in and knowledgeable about the topic as compared with the general population. If that were the case, these findings further underscore women’s need for information. Another limitation is that we were not able to explore differences across the myriad groups that constitute the three major racial/ethnic groups included in this study, for example by region or country of origin. We recognize the tremendous heterogeneity within these groups, but resource limitations prohibited from segmenting focus groups on these factors.

Despite these limitations, the focus groups approach enabled us to identify participant-driven themes, illuminating variations in perspectives. The data are useful in generating hypotheses that can be tested in future research. Moreover, these findings provide insights for the development of interventions to address the recent guideline change.

#### ***Intervention opportunities***

Awareness of guideline changes was low in this sample, so targeted efforts to increase knowledge and awareness of changes in mammography recommendations are needed. However, simply increasing knowledge and awareness will not be sufficient. Our findings suggest that contextual factors – including community and cultural norms, as well as issues of medical mistrust and fear – need to be addressed when conveying changes in screening guidelines, particularly if recommendations call for less (not more) screening. Changing social norms about breast cancer screening may require undoing much of the health messaging that has been delivered over the past several decades. Numerous studies document the fact that women overestimate their risk of breast cancer [56,57] and underestimate the fallibility of mammography [58]. Since prior research shows that social norms and perceptions about the importance of mammography to family members can affect screening practices [59-63], interventions are needed to normalize the idea that one might *choose* to forgo cancer screening in some circumstances. This will require quite a bit of ‘undoing’ prior public health campaigns that stressed “get a mammogram, once a year for life”. In such a context, hearing similar age peers, family members or role models talk about their reasons for choosing not to undergo screening at a young age could help others to see that this could be a reasonable choice, given the tests’ limitations and potential for adverse outcomes. Moreover, since screening guidelines are likely to change in the future as new technologies are tested and put into practice, a focus on helping women to become more “informed consumers” of health information might be a more important intervention goal, rather than instilling the directive from prior campaigns: “Get a mammogram”.

Many women expressed that they preferred to receive information about breast cancer screening directly from their providers, although there were some differences observed across racial/ethnic groups. Latinas frequently reported that they relied on female family members for health advice, but many also reported that they “followed doctor’s orders” without question. Among African American women, mistrust of provider motives was more often discussed, and with this came with the perception that women need to advocate for themselves by preparing themselves with information. Although many of the Caucasian women also expressed a belief in the importance of shared decision-making, none reported having a specific positive experience with shared decision-making. Experiences of poor patient-provider communication were frequently reported across all of the groups, and many women were pessimistic about the idea that medical decisions could truly be shared.

Our original plans for this formative research had been to use the results to develop a decision aid for women ages 40–50 so that they would be better informed about mammography screening, and therefore, more prepared to participate in shared-decision making. However, our findings suggest that women do not see the relevance of decision aids in the context of breast cancer screening. For the most part, women did not see that there was a decision to be made; they simply wanted to be screened for breast cancer. However, as most women wanted more information about breast cancer, yet eschewed decision aids (perhaps due to lack of full understanding of their educational nature), other types of targeted communication efforts may be needed to improve patient-provider communication. Contextualizing breast cancer outreach and resources in light of cultural considerations may support acceptance and participation in informed decision-making activities [64]. The prominence of self-advocacy in our discussion themes also provides an opportunity to consider ways of positioning judicious use of mammography as something that may be the most beneficial course of action.

Interventions to equip women with the information they need to make informed decisions, as well as dyadic interventions to improve communication skills between patients and providers are needed. The latter is a particular challenge, as time for a routine medical visit averages just 21 minutes in U.S. primary care practices [65]. Use of e-Health technology, including multi-media educational tools, can help convey complicated messages without taking time away from the visit [66]. In similar research related to prostate cancer screening, simple informational tools, such as fact boxes that list out screening harms and benefits, were suggested for communicating screening decisions [67]. More research is needed to assess how communication tools can be used by patient navigators or outreach workers, who provide direct breast health information and often influence women’s decisions about screening [68].

Women also suggested that making decision support or educational tools available outside of clinical settings, for example, in familiar community settings, would be useful. A number of African American women advised sharing information in locations they visited regularly, such as churches, grocery/corner stores, libraries, laundro-mats, schools and health clinics. Latinas agreed and suggested social settings frequented by members of their communities, including local restaurants and food stores. Distribution of information in socially relevant community settings has been found to be successful among diverse audiences [69-71], and should continue to be a part of comprehensive breast cancer control efforts.

### Conclusion

Future studies with larger samples of ethnically diverse women are needed to verify these findings and could identify themes specifically related to cultural influences on attitudes, beliefs, and knowledge. Additionally, since prior research has suggested that knowledge and uptake of screening decision aids may vary with SES and other characteristics [72], a comparison with women of varying SES levels could be useful. Additional research that evaluates the short- and long-term effects of guideline changes, including women’s adherence to screening guidelines and provider practices/recommendations, as well as breast cancer morbidity and mortality, is needed. As the evidence base for breast cancer screening continues to grow and as new technologies become available, it is likely that screening recommendations will continue to evolve. Given that change is inevitable, preparing women to be informed consumers of information regarding the efficacy of available screening modalities and providing them with skills to make informed decisions with their health care providers should be a primary goal for cancer control interventions.

### Consent

We confirm that all patient/personal identifiers have been removed or disguised so the patient/person(s) described are not identifiable and cannot be identified through the details of the story.

## Competing interests

The authors declare that they have no competing interests.

## Authors’ contributions

JA conceptualized the study, oversaw the design, implementation and data analysis, and drafted the initial manuscript. SB collaborated on data analysis, contributed to literature review and drafted sections of the manuscript. MS collaborated on data analysis and summarization. KO, KG, and EH collaborated on development of data collection instruments and the analysis of findings, and also facilitated collaboration with the Avon-funded community based organizations to conduct the focus groups. MH contributed essential intellectual content. All authors read and approved the final manuscript.

## Pre-publication history

The pre-publication history for this paper can be accessed here:

http://www.biomedcentral.com/1471-2458/13/1169/prepub
